# Characteristics of Growth, Photosynthesis, C/N Ratio, and Antioxidant Capacity in the Seedling Stage of *Aquilaria sinensis* ‘Qinan’

**DOI:** 10.3390/plants14060896

**Published:** 2025-03-13

**Authors:** Qilei Zhang, Ning Ma, Yu Su, Xiaojin Liu

**Affiliations:** 1 Research Institute of Tropical Forestry, Chinese Academy of Forestry, Guangzhou 510520, China; dalei45666@163.com (Q.Z.);; 2 State Key Laboratory of Efficient Production of Forest Resources, Beijing 100091, China; 3 College of Landscape Architecture, Nanjing Forestry University, Nanjing 210037, China; 4 Guangzhou Institute of Forestry and Landscape Architecture, Guangzhou 510080, China; 5 Guangzhou Collaborative Innovation Center on Science-Tech of Ecology and Landscape, Guangzhou 510405, China

**Keywords:** antioxidant, *Aquilaria sinensis*, C/N ratio, leaf area, photosynthesis

## Abstract

In this study, *Aquilaria sinensis* ‘Qinan’, a strain of *A. sinensis* that easily forms agarwood, was selected and propagated by grafting seedlings. Existing research has mainly focused on the characteristics of agarwood formation, but little attention has been paid to the growth characteristics of *A. sinensis* ‘Qinan’. In this study, the growth rate, photosynthetic capacity, leaf size, carbon and nitrogen contents, and antioxidant capacity were evaluated during the early growth stage in *A. sinensis* ‘Qinan’ and *A. sinensis* grafted seedlings. Compared with *A. sinensis*, *A. sinensis* ‘Qinan’ exhibited higher net photosynthetic rate (9.2 μmol m^−2^ s^−1^ in *A. sinensis* ‘Qinan’ and 7.8 μmol m^−2^ s^−1^ in *A. sinensis*) in the mature leaf. There were higher contents of secondary metabolites such as flavonoids and phenols, with stronger antioxidant capacity in *A. sinensis* ‘Qinan’. Larger leaf area (43.9 cm^2^ in *A. sinensis* and 30.1 cm^2^ in *A. sinensis* ‘Qinan’), higher nitrogen content (24.9 mg kg^−1^ in *A. sinensis* and 23.7 mg kg^−1^ in *A. sinensis* ‘Qinan’) in young leaves, faster growth rate, and larger biomass were observed in *A. sinensis*. The results indicate that differences exist in nutrient distribution during the growth process of *A. sinensis* ‘Qinan’ and *A. sinensis*, with more substances being used to synthesize defensive secondary metabolites in *A. sinensis* ‘Qinan’.

## 1. Introduction

Agarwood is a mixture formed by the secretions and woody tissue of genus *Aquilaria* trees after external injury [1]. Due to its wide application, high value, and low natural yield, agarwood is in short supply [2,3]. At present, the genus *Aquilaria* has been listed as a protected plant. In order to increase the supply of agarwood, large-scale artificial cultivation of genus *Aquilaria* trees has been undertaken [4].

The formation of agarwood is a biochemical process that is related to various factors [5,6,7], and ordinary *Aquilaria sinensis* trees have a slow and low rate of agarwood formation [8]. At present, a new type of *A. sinensis* germplasm (*A. sinensis* ‘Qinan’) that easily forms agarwood has been discovered, exhibiting rapid agarwood formation and a high agarwood yield [9]. Its characteristic of easy agarwood formation can be preserved through grafting [1].

*A. sinensis* ‘Qinan’ forms agarwood earlier in the growth stage than *A. sinensis*. At approximately 10 years of age, *A. sinensis* trees are used for inducing agarwood, while *A. sinensis* ‘Qinan’ trees only need to grow for 3 years before use for agarwood production [1,8]. During the process of forming agarwood in *A. sinensis*, it is often necessary to drill fire holes, add salt after drilling, or hang reagents, while simply drilling cold drills can cause *A. sinensis* ‘Qinan’ to produce agarwood [8]. During the process of agarwood formation, the *A. sinensis* trees undergo a stress response due to damage, resulting in an increase in the content of reactive oxygen species in the body [10]. The stress response of *A. sinensis* ‘Qinan’ is stronger, with stronger antioxidant enzyme activity than that of *A. sinensis*. The content of secondary metabolites such as total phenols, salicylic acid, and terpenes in *A. sinensis* ‘Qinan’ are significantly higher than those in *A. sinensis* [1]. During the process of agarwood formation, the starch content in the stem gradually decreases, while the soluble sugar content first increases and then decreases. The changes in starch and soluble sugar content in *A. sinensis* ‘Qinan’ are significantly greater than those in *A. sinensis* [1]. Moreover, the yield of *A. sinensis* ‘Qinan’ is greater than that of *A. sinensis*. After 6 months of inducing agarwood, the agarwood yield and oil content of *A. sinensis* ‘Qinan’ exceeded 38% and 24%, respectively, while the agarwood yield and oil content of *A. sinensis* were below 6% and 8%, respectively [1].

Many studies have focused on the quality differences between *A. sinensis* ‘Qinan’ and *A. sinensis*, whereas there are few reports on the growth characteristics of the seedling stage of *A. sinensis* ‘Qinan’. In this study, *A. sinensis* ‘Qinan’ and *A. sinensis* grafted seedlings were used as materials to analyze the physiological differences during the seedling stage. The results provide a scientific basis for the early selection and breeding of excellent varieties of *A. sinensis* ‘Qinan’.

## 2. Results

### 2.1. Growth

**Figure 1:**
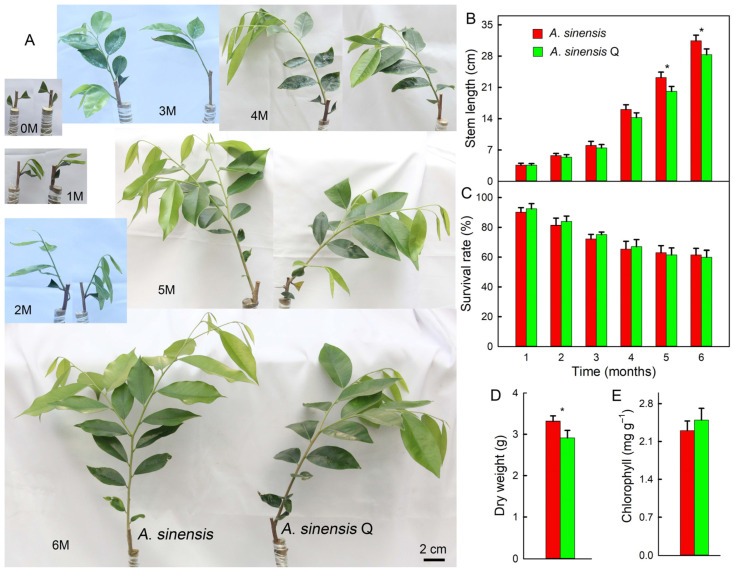
Phenotype (**A**), stem length (**B**), and survival rate (**C**) changes in *Aquilaria sinensis* (*A. sinensis*) and *Aquilaria sinensis* ‘Qinan’ (*A. sinensis* Q) from 0 to 6 months (0 M–6 M). Dry weight (**D**) and chlorophyll content (**E**) after 6 months. Student’s *t*-test analysis indicates a significant difference (* *p* < 0.05).

After 0–6 months (0 M–6 M) of grafting, differences were observed in the growth of *A. sinensis* ‘Qinan’ (*A. sinensis* Q) and *A. sinensis* (Figure 1A,B). The growth rate of *A. sinensis* was faster than *A. sinensis* ‘Qinan’, and its stem length was significantly higher than *A. sinensis* ‘Qinan’ after 5 months. The survival rate of *A. sinensis* and *A. sinensis* ‘Qinan’ gradually decreased after grafting, with no significant difference shown between the two (Figure 1C). After 6 months, the dry weight of *A. sinensis* was significantly higher than that of *A. sinensis* ‘Qinan’ (Figure 1D). The chlorophyll content in the leaves of *A. sinensis* ‘Qinan’ was higher; however, the difference in leaf chlorophyll content between *A. sinensis* and *A. sinensis* ‘Qinan’ was not significant (Figure 1E).

### 2.2. Gas Exchange Parameters

**Figure 2:**
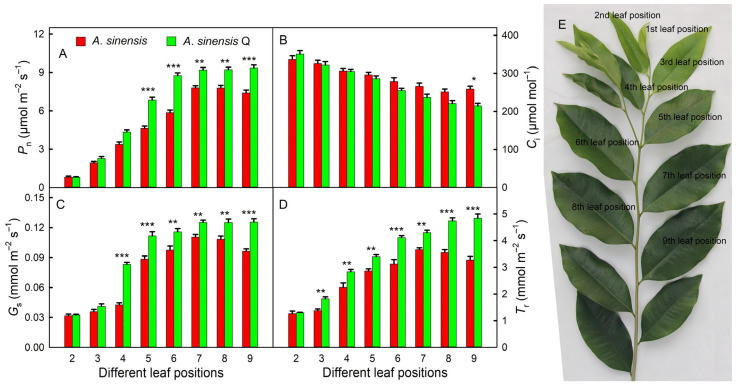
Net photosynthetic rate (*P*_n_, **A**), intercellular CO_2_ concentration (*C*_i_, **B**), stomatal conductance (*G*_s_, **C**), and transpiration rate (*T*_r_, **D**) in the leaves of *Aquilaria sinensis* (*A. sinensis*) and *Aquilaria sinensis* ‘Qinan’ (*A. sinensis* Q) at different leaf positions (**E**). Student’s *t*-test analysis indicates a significant difference (* *p* < 0.05; ** *p* < 0.01; *** *p* < 0.001).

As the leaves matured, the net photosynthetic rate (*P*_n_) gradually increased. The *P*_n_ at the sixth leaf position of *A. sinensis* ‘Qinan’ was close to the maximum value, and the *P*_n_ of the sixth, seventh, eighth, and ninth leaf positions was similar (the different leaf positions were shown in Figure 2E). The *P*_n_ at the seventh leaf position of *A. sinensis* was close to the maximum value, while the *P*_n_ at the ninth leaf position decreased. Starting from the fifth leaf position, the *P*_n_ of the *A. sinensis* ‘Qinan’ leaves was significantly higher than that of *A. sinensis* (Figure 2A). The intercellular CO_2_ concentration (*C*_i_) gradually decreased with the maturity of the leaves. Starting from the sixth leaf position, the *C*_i_ in the leaves of *A. sinensis* ‘Qinan’ was lower than that of *A. sinensis* and the *C*_i_ in the ninth leaf position of *A. sinensis* ‘Qinan’ was significantly lower than that of *A. sinensis* (Figure 2B). The trend of change in stomatal conductance (*G*_s_) and transpiration rate (*T*_r_) was consistent with the trend of change in *P*_n_. The *G*_s_ of *A. sinensis* ‘Qinan’ was significantly higher than that of *A. sinensis* from the fourth leaf position, and the *T*_r_ was significantly higher than that of *A. sinensis* from the third leaf position (Figure 2C,D).

### 2.3. Light Response Curves

**Figure 3:**
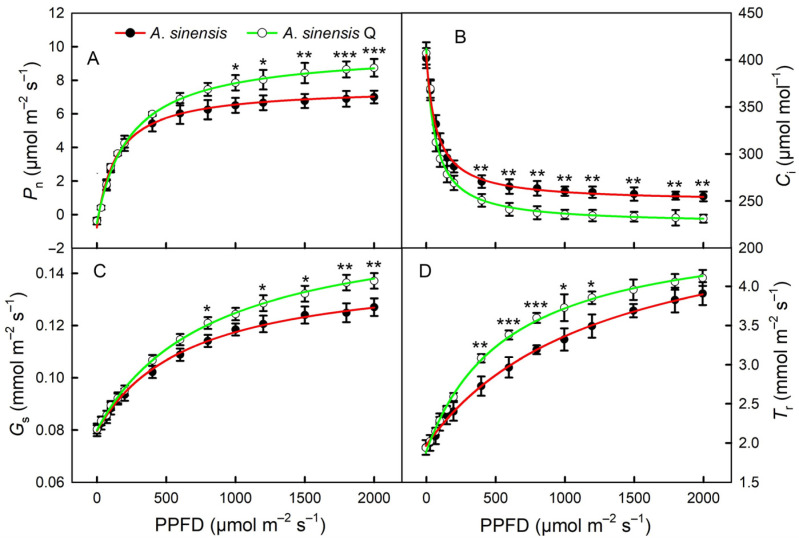
Light response curves of net photosynthetic rate (*P*_n_, **A**), intercellular CO_2_ concentration (*C*_i_, **B**), stomatal conductance (*G*_s_, **C**), and transpiration rate (*T*_r_, **D**) in the leaves of *Aquilaria sinensis* (*A. sinensis*) and *Aquilaria sinensis* ‘Qinan’ (*A. sinensis* Q) at the eighth leaf position. PPFD, photosynthetic photon flux density. Student’s *t*-test analysis indicates a significant difference (* *p* < 0.05; ** *p* < 0.01; *** *p* < 0.001).

As the light intensity increased, the *P*_n_ gradually increased. After the light intensity reached more than 600 μmol m^−2^ s^−1^, the *P*_n_ of *A. sinensis* ‘Qinan’ leaves was higher than that of *A. sinensis* (Figure 3A). The *C*_i_ gradually decreased with increasing light intensity, and the *C*_i_ in the leaves of *A. sinensis* ‘Qinan’ was lower than that of *A. sinensis* when the light intensity was higher than 400 μmol m^−2^ s^−1^ (Figure 3B). The trend of change in *G*_s_ and *T*_r_ was consistent with the trend of change in *P*_n_ (Figure 3C,D).

### 2.4. Leaf Parameters, C/N Ratio, and Antioxidant Capacity

**Figure 4:**
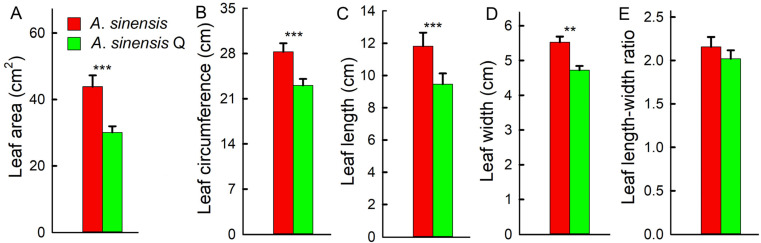
Leaf area (**A**), leaf circumference (**B**), leaf length (**C**), leaf width (**D**), and the leaf length–width ratio (**E**) of *Aquilaria sinensis* (*A. sinensis*) and *Aquilaria sinensis* ‘Qinan’ (*A. sinensis* Q). Student’s *t*-test analysis indicates a significant difference (** *p* < 0.01; *** *p* < 0.001).

The leaf area, leaf length, leaf width, and leaf circumference of *A. sinensis* ‘Qinan’ were significantly smaller than those of *A. sinensis*. There was no significant difference in the leaf length–width ratio between *A. sinensis* ‘Qinan’ and *A. sinensis* ‘Qinan’ (Figure 4).

**Figure 5:**
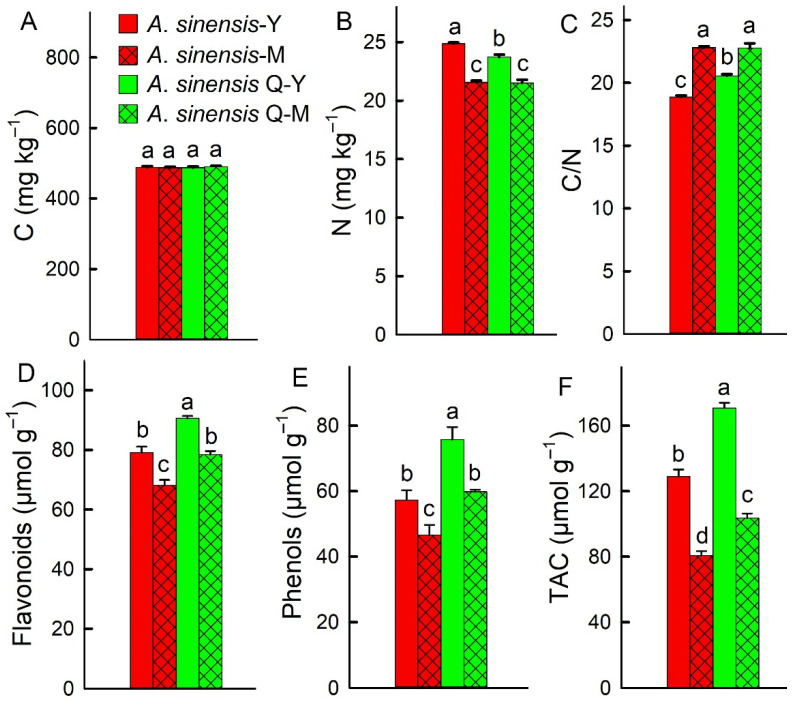
The carbon (C, **A**), nitrogen (N, **B**), C/N (**C**), flavonoids (**D**), phenols (**E**), and total antioxidant capacity (TAC, **F**) in the young leaves of *Aquilaria sinensis* (*A. sinensis*-Y), the mature leaves of *A. sinensis* (*A. sinensis*-M), the young leaves of *A. sinensis* ‘Qinan’ (*A. sinensis* Q-Y), and the mature leaves of *A. sinensis* ‘Qinan’ (*A. sinensis* Q-Y). Different letters above the bars indicate significant differences (*p* < 0.05).

There was no significant difference in carbon (C) content between the young and mature leaves of *A. sinensis* ‘Qinan’ and *A. sinensis* (Figure 5A). The nitrogen (N) content in the young leaves of *A. sinensis* ‘Qinan’ was significantly lower than that of *A. sinensis*. There was no significant difference in N content between the mature leaves of *A. sinensis* ‘Qinan’ and *A. sinensis*, which was significantly lower than that in the young leaves (Figure 5B). There was no significant difference in the C/N ratio content between the mature leaves of *A. sinensis* ‘Qinan’ and *A. sinensis*, which was significantly higher than that of the young leaves. The C/N ratio in the young leaves of *A. sinensis* ‘Qinan’ was significantly higher than that of *A. sinensis*, with the lowest C/N ratio observed in the young leaves of *A. sinensis* (Figure 5C).

The contents of flavonoids and phenols in the young leaves of *A. sinensis* ‘Qinan’ were the highest, which were significantly higher than that in the mature leaves of *A. sinensis* ‘Qinan’ and the leaves of *A. sinensis*. There was no significant difference in flavonoids and phenols between the mature leaves of *A. sinensis* ‘Qinan’ and the young leaves of *A. sinensis*. The content of flavonoids and phenols in the mature leaves of *A. sinensis* were the lowest, which were significantly lower than that in the young leaves of *A. sinensis* and the leaves of *A. sinensis* ‘Qinan’ (Figure 5D,E). The total antioxidant capacity (TAC) in the young leaves of *A. sinensis* ‘Qinan’ was the highest, which was significantly higher than that of the *A. sinensis* ‘Qinan’ mature leaves and the *A. sinensis* leaves. The TAC in the young leaves of *A. sinensis* was significantly higher than that of the *A. sinensis* mature leaves and *A. sinensis* ‘Qinan’ leaves. The TAC in the mature leaves of *A. sinensis* ‘Qinan’ was significantly higher than that of *A. sinensis*, and the TAC of the *A. sinensis* mature leaves was the lowest (Figure 5F).

## 3. Discussion

The results of the experiment conducted in this study indicate that the photosynthetic capacity of *A. sinensis* ‘Qinan’ leaves was stronger than that of *A. sinensis*. Photosynthesis is the foundation of plant growth, and chlorophyll plays a key role in the reaction process, which directly affects the net photosynthetic rate [11,12]. The results show that the chlorophyll content in the leaves of *A. sinensis* ‘Qinan’ was higher than that of *A. sinensis*, which is consistent with the results of the net photosynthetic rate. The photosynthetic capacity of mature leaves is stronger than young leaves. This may be because incomplete development of stomata on young leaves directly affects gas exchange and lower stomatal conductance leads to reduced photosynthetic capacity [13]. The net photosynthetic rate of *A. sinensis* ‘Qinan’ was higher than that of *A. sinensis* in the same leaf position. This finding may be due to the chlorophyll being positively correlated with photosynthetic capacity in the leaves [13].

The leaves of *A. sinensis* were significantly larger than that of *A. sinensis* ‘Qinan’. Leaves are the main organs for photosynthesis in plants, and leaf size directly affects the plant’s ability to capture light energy and total photosynthetic capacity [14]. Leaves are one of the organs most sensitive to environmental changes, and their phenotypic characteristics have great plasticity. There are differences in leaf phenotypes between different species or different families of the same substance [15,16,17]. Increasing leaf area is an important strategy for trees to obtain more light and accelerate their own growth [18,19,20]. In this study, the larger leaf area of *A. sinensis* provided a prerequisite for the accumulation of photosynthetic products. In this study, the leaf area of *A. sinensis* were significantly larger than those of *A. sinensis* ‘Qinan’, which led to the biomass being significantly higher in *A. sinensis*. Previous studies have found that larger leaf areas promoted biomass accumulation [14]. It has been found that crop and fruit yields, as well as tree biomass, were positively correlated with leaf area [21,22,23]. The results suggest that *A. sinensis* obtained more light energy by increasing leaf area, improved total photosynthetic products, and promoted growth.

The contents of flavonoids and phenols were higher in *A. sinensis* ‘Qinan’, and the antioxidant capacity of *A. sinensis* ‘Qinan’ was stronger than *A. sinensis*. This is related to the characteristic of easier agarwood formation in *A. sinensis* ‘Qinan’, and the antioxidant capacity of *A. sinensis* ‘Qinan’ agarwood is stronger, with a higher content of antioxidant substances [1,8]. Flavonoids and phenols are non-enzymatic antioxidants in plants, and their contents are positively correlated with antioxidant capacity [24]. Under stress conditions, the growth of plants is restricted, and the levels of flavonoids and phenols in their bodies significantly increase, enhancing the antioxidant capacity [25,26,27]. In this study, the content of flavonoids and phenols in young leaves were significantly higher than that in mature leaves, and the antioxidant capacity was significantly stronger than that in mature leaves. This may be due to the underdeveloped photosystem of young leaves requiring less light energy; however, young leaves receive the same light intensity as mature leaves during growth. Excessive light causes greater damage to young leaves; thus, more antioxidants are needed to provide protection [24]. This is consistent with the research results of other scholars, who found that the content of chlorophyll in young leaves was lower, the photosynthetic capacity was weaker, and young leaves were more susceptible to light damage [13]. Young leaves reduce the damage caused by high light by increasing antioxidant substances such as flavonoids and phenols or by accumulating colored anthocyanins to reduce the absorption of light energy [28]. The flavonoid and phenol contents of *A. sinensis* ‘Qinan’ were significantly higher than that of *A. sinensis*; moreover, the antioxidant capacity of *A. sinensis* ‘Qinan’ was stronger than that of *A. sinensis*. This may be related to the characteristic of easy agarwood formation in *A. sinensis* ‘Qinan’ [1]. Previous studies have found that, after being wounded, *A. sinensis* trees form agarwood, which contains flavonoids and antioxidant properties [27,29,30]. The stress response of *A. sinensis* ‘Qinan’ was stronger after being wounded, with significantly more antioxidant substances and stronger antioxidant enzyme activity than that of *A. sinensis* [1]. Moreover, the content of flavonoids and phenols in the essential oil of *A. sinensis* ‘Qinan’ was significantly higher than that of *A. sinensis* [1]. This indicates that more defensive substances were accumulated in *A. sinensis* ‘Qinan’ before being wounded.

There was no significant difference in carbon (C) content between *A. sinensis* ‘Qinan’ and *A. sinensis*, but there were significant differences in nitrogen (N) content and the C/N ratio. The C/N ratio reflects the allocation of nutrients in plants [31]. Generally, more N will be supplied to young leaves during growth and development, while reducing N suppling in mature and aging leaves [32,33]. Previous studies have found that the N content in young leaves was significantly higher than that in mature leaves [34], which is consistent with the results of this study. The results of this study show that the N content in the young leaves was significantly higher than that in mature leaves. In this study, the N content in the young leaves of *A. sinensis* was significantly higher than that of *A. sinensis* ‘Qinan’. More N in the young leaves of *A. sinensis* can promote their growth. It was found that there were significant differences in the N content of different plant leaves, with higher N content in the more vigorous growing young leaves [32]. This result indicates that the growth and renewal rate of *A. sinensis* young leaves is faster than that of *A. sinensis* ‘Qinan’, which is consistent with the results of stem length changes and biomass. Studies have found that, as the leaves matured, the N content in the leaves gradually decreased [35]. There was no significant difference in N content between the mature leaves of *A. sinensis* ‘Qinan’ and *A. sinensis*. Studies on the two plants found that, although there was significant difference in the N content of the young leaves, there was no significant difference in the N content of the mature leaves [32]. This is consistent with the N content in mature leaves of *A. sinensis* ‘Qinan’ and *A. sinensis* in this study.

The growth rate of *A. sinensis* ‘Qinan’ was slower than that of *A. sinensis*. As the basis for plant material synthesis and accumulation, the rate of photosynthesis directly affects plant growth and development. The results show that the net photosynthetic rate of the *A. sinensis* ‘Qinan’ leaves was significantly higher than that of *A. sinensis*, but that its stem length and biomass were significantly lower than those of *A. sinensis*. This may be due to more substances being used to synthesize defensive secondary metabolites such as flavonoids and phenols, enhancing antioxidant capacity and accumulating antioxidant substances in advance for later stress responses in *A. sinensis* ‘Qinan’ [1]. In addition, more nutrients were supplied in young leaves to promote their development and renewal in *A. sinensis*, thereby forming more leaves for photosynthesis and promoting growth [32,36]. Furthermore, the leaves of *A. sinensis* were larger, which increases the total photosynthetic products [36]. Although these physiological indicators of *A. sinensis* ‘Qinan’ are different from *A. sinensis*, they cannot be used as a direct basis for screening *A. sinensis* ‘Qinan’ in the seedling stage. In the future, it will be necessary to further distinguish them using molecular technology.

## 4. Materials and Methods

### 4.1. Plant Materials

The experiment was conducted in late October 2023 at the Research Institute of Tropical Forestry, Chinese Academy of Forestry (longitude 113.38.47 E, latitude 23.19.07 N), Guangzhou, China. Healthy one-year-old seedlings of *A. sinensis* with a ground diameter of 1.3–1.6 cm were used as rootstocks, and one-year-old branches of *A. sinensis* and *A. sinensis* ‘Qinan’ were used as scions. *A. sinensis* and *A. sinensis* ‘Qinan’ were grafted with 200 plants each, for a total of 400 plants, divided into 5 groups, with each group containing 40 grafted seedlings of *A. sinensis* and 40 grafted seedlings of *A. sinensis* ‘Qinan’. The phenotype, survival rate, and stem length were recorded every month after grafting, and other physiological indicators were measured at 6 months.

### 4.2. Gas Exchange, Leaf Parameters, and Chlorophyll Measurement

The gas exchange parameters of the leaves were determined at different positions (1–9) from 9:00 to 11:00 on sunny days according to Zhang et al. [27] using the LI-6800 system (LI-COR, Lincoln, NE, USA). The light intensity in the detection chamber was 1100 μmol m^–2^ s^–1^, the ratio of red to blue light was 9:1, and the CO_2_ concentration was 400 μmol mol^–1^. Gas exchange parameters were recorded after the value was relatively stable.

Photosynthesis light response curves were measured for the leaves at the eighth leaf position with fourteen irradiances (0–2000 μmol m^−2^ s^−1^). The ratio of red to blue light was 9:1, and the CO_2_ concentration was 400 μmol mol^−1^. The waiting times were 90–180 s for each irradiance [13].

A leaf-area meter CI-203 (CID, Camas, WA, USA) was used to measure the leaf parameters of the eighth leaf position.

The chlorophyll was extracted from 0.05 g of fresh leaves (eighth leaf position) in 6 mL of 80% acetone. The extracts were used to detect the chlorophyll content according to Wellburn [37].

### 4.3. Carbohydrate and Nitrogen Content Measurement

Dry leaves were ground into powder, and 0.3 g of leaf powder was accurately weighed. A 15 mL centrifuge tube containing 8 mL of 80% ethanol was used to collect the leaf powder. The centrifuge tube containing the sample was subjected to an 80 °C water bath for 30 min and then centrifuged at 5000× *g* for 10 min. The supernatant was collected and diluted with deionized water to 25 mL. The soluble sugars were determined using the anthrone reagent, according to the method described by Yemm and Willis [38]. The ethanol-insoluble residue was extracted for starch and measured using the anthrone reagent, according to the method described by Clegg [39]. Total non-structural carbohydrates were calculated as the sum of soluble sugars and starch.

The leaf powders (0.15 g) were put into a 50 mL digestion tube and digested with 5 mL concentrated (98%) H_2_SO_4_ at 180 °C. Nitrogen content was determined using the standard macro-Kjeldahl procedure using the Kjeltec 2300 analyzer (FOSS, Copenhagen, Denmark).

### 4.4. Flavonoids, Phenols, and Total Antioxidant Capacity Measurement

The 0.05 g leaf was extracted in 1.5 mL of 95% methanol at 4 °C for the determination of flavonoids, phenols, and total antioxidant capacity according to Zhang et al. [13]. Different concentrations of catechins were used to create standard curves for calculating flavonoid concentration at 510 nm. Different concentrations of gallic acid were used to create standard curves for calculating phenol concentration at 765 nm. Different concentrations of 1,1-diphenyl-2-picrylhydrazyl (DPPH) were used to create standard curves for calculating total antioxidant capacity at 517 nm.

### 4.5. Statistical Analysis

Student’s *t*-test, one-way analysis of variance (ANOVA), and Duncan’s post hoc test were used for statistical significance analysis using SPSS Statistics 19.0 (IBM, Armonk, NY, USA) at the level of 0.05. SigmaPlot 12.5 (Systat Software Inc., San Jose, CA, USA) was used to plot the data.

## 5. Conclusions

This study reveals differences in the growth of *A. sinensis* ‘Qinan’ and *A. sinensis* grafted seedlings during the seedling stage. In *A. sinensis* ‘Qinan’, the content of chlorophyll was higher and the photosynthetic capacity was stronger; moreover, the contents of flavonoids and phenolic secondary metabolites were higher and the antioxidant capacity was stronger in *A. sinensis* ‘Qinan’. In *A. sinensis*, the larger leaf area and higher N content in the young leaves increased the growth rate and biomass. Therefore, in the process of breeding *A. sinensis* ‘Qinan’, priority should be given to selecting plants with strong photosynthetic and antioxidant abilities and smaller leaf areas during the seedling stage.

## Data Availability

The original contributions presented in this study are included in the article. Further inquiries can be directed to the corresponding authors.
